# Aggregates Obtained by Alkali Activation of Fly Ash: The Effect of Granulation, Pelletization Methods and Curing Regimes

**DOI:** 10.3390/ma12050776

**Published:** 2019-03-06

**Authors:** Ognjen Rudić, Vilma Ducman, Mirjana Malešev, Vlastimir Radonjanin, Suzana Draganić, Slobodan Šupić, Miroslava Radeka

**Affiliations:** 1 AD “Polet” IGK, Factory “Stražilovo”, Sremski Karlovci, 35 Krivac, Serbia; ognjen.rudic@nexe.rs; 2 National Building and Civil Engineering Institute, Dimičeva ulica 12, SI-1000 Ljubljana, Slovenia; vilma.ducman@zag.si; 3 Department of Civil Engineering, Faculty of Technical Sciences, University of Novi Sad, Trg Dositeja Obradovica 6, 21000 Novi Sad, Serbia; miram@uns.ac.rs (M.M.); radonv@uns.ac.rs (V.R.); suzanav@uns.ac.rs (S.D.); ssupic@uns.ac.rs (S.Š.)

**Keywords:** fly ash, alkali activation, aggregate, crushing method, pelletization

## Abstract

This paper presents results regarding the phase composition, microstructure and textural properties of two types of aggregates, which were prepared via crushing or pelletization of alkali-activated Class F fly ash and cured under different conditions. The alkali activator was the same for aggregate products, containing an alkaline solution consisting of 8 M NaOH and Na-silicate (8 M NaOH/Na-silicate = 1:2.5 mass ratio). The aforementioned properties were influenced by two different preparation procedures combined with varying curing regimes (under normal conditions at 20 °C, RH 40–60% for 28 and 120 days and under an accelerated regime, at 65 °C for 5 days). Aggregates were characterized using X-ray diffraction (XRD), Fourier-transform transmission infrared spectroscopy (FTIR), back scattered electron microscopy with energy dispersive spectrometer (BSE-EDS) analyses and mercury intrusion porosimetry (MIP). The results showed noteworthy structural and textural diversities between the two types of aggregate. The method of preparation and curing regime affected the formation of the N-A-S-H structure and the texture of the alkali-activated fly ash product, with the crushing method giving an advantage.

## 1. Introduction

Concrete, as one of the most widespread construction materials, has a great environmental impact, since it contributes to air pollution and consumes natural materials [1]. Analyses have shown that the production of cement, the main component of concrete, consumes a great amount of energy and is regarded as the main culprit for the release of greenhouse gases, while the consumption of the second most important component, aggregate, requires mining billions of tons of natural aggregate [2]. There is a lack of high quality natural aggregate in some parts of the world, for instance, aggregate resources in the United Kingdom are being consumed twice as fast as they can be replenished [3]. In recent years, an increasing interest in the replacement of natural aggregates with artificial ones made of waste materials presents an important environmental issue. If the starting material is solid material, the common techniques for the production of coarse, artificial (recycled) aggregates are crushing and grinding. Recycled aggregate can be used for: recycled concrete [4], sanitary ceramic wastes [5], brick ceramic waste [6], glass wastes [7], and plastic wastes [8]. Research dedicated to the use of fly ash, as a powder material, in the production of coarse aggregates has therefore become crucial, as the global production of coal will rise by over 50% by 2030 and consequently the production of fly ash will also increase [9]. The production of coarse aggregate from powdered material can be realized by the agglomeration of fines. This can be achieved (i) through the process of pelletization (on granulating plates) [10] or (ii) by pouring a mixture of fly ash and a binder into molds; the hardened samples are then crushed and sieved in order to obtain aggregate. The products of pelletization are pellets, formed by adding a wetting agent (water, different types of binders) to the powdered material [11] prior and/or during pelletization. When an alkali activator is used as a binder (wetting agent), the chemical reaction between the binder and fly ash, in addition to wetting, influences the formation of granules (fresh pellets) during pelletization. Several factors should be taken into account in order to study the complex influence of alkali activation on the microstructural and textural properties of aggregate pellets. The first factor is the inhomogeneity of the alkali activation process. Under these circumstances, the alkali activator might not be distributed evenly throughout all granules. The second factor, water evaporation, might be more intensive in the initial phase of alkali activation in pelletization than when using the crushing method. On the other hand, since the conditions in which alkali activation occurs during pelletization are different from those experienced in the crushing method, the paths of alkali activation are also diverse.

Generally, microstructural changes during alkali activation of the starting material are the result of a series of specific destruction–condensation reactions [12]. The dissolution of reactive aluminosilicate from the amorphous phase in the presence of a high concentration of OH^−^ (basic pH) is expressed as the dissolution of Si^4+^ and Al^3+^ ions and the formation of monomeric groups in the solution. If the dissolution of aluminosilicates is fast, which depends on the fly ash composition [13], the solution is quickly over-saturated. The oligomers made by the interaction of monomeric groups therefore condensate and form a Gel 1 structure (N-A-S-H gel). This gel is rich in aluminum, as Al–O bonds are weaker than Si–O bonds. The process of aluminosilicate dissolution via alkaline hydrolysis consumes water. A lack of water during this stage of alkali activation therefore retards dissolution [14].

Over time, more Si^4+^ ions dissolve and their concentration in the solution becomes higher [15], influencing the reorganization of Gel 1 and thus enabling the formation of Gel 2 (N-A-S-H precipitation) through the polycondensation of dissolved species. Gel 2 represents a 3D network formed of silico–aluminate structures, comprising structures with Si–O–Si and Si–O–Al bonds. As Si–O–Si bonds are stronger than Si–O–Al or Al–O–Al bonds, a gel structure with a higher Si/Al atomic ratio value will have a stronger structure and exhibit better mechanical and physical properties [16]. According to the assumed structures of Gel 1 and Gel 2, it is evident that the Si/Al atomic ratio value should be higher in Gel 2 than in Gel 1, and determination of its value can therefore be used as a criterion to follow the progress of the structural transformation of Gel 1 into Gel 2. During the gelation process and the formation of Gel 2, water, which is necessary for diffusion during dissolution, is released. Some of this water fills the pores of the gel, while the rest should be eliminated by evaporation, indicating the importance of the curing regime during alkali activation [16].

In light of the above facts, some important issues will be discussed regarding the parameters that influence these processes during the production of aggregate by granulation—either via crushing or by pelletization. Both methods are likely to have an uneven distribution of alkali activator, which could change the alkali activator/fly ash ratio compared to the desired values; pelletization, however, might also provide the possibility of faster granule drying, i.e., loss of water. These occurrences, along with other factors (the impact of carbonation and calcium ion on the alkali activation) still to be discussed, might affect the quality of the N-A-S-H gel, i.e., the Si/Al ratio [17].

Increased alkali content may be evident in certain parts of the reactant fly ash mixture due to the inhomogeneity of alkali activator distribution. When alkali-activated samples are exposed to the atmosphere, excess alkali could be involved in the formation of carbonates, having a deleterious effect on the properties of the material. Carbonation is most effective when the relative humidity is between 50 and 70% [18] because the diffusion rate of CO_2_ is 10 times greater in air than in water [16]. It is obvious that the process of carbonation occurs simultaneously with alkali activation if the curing conditions are convenient for carbonization. According to Bernall et al. [19], the carbonation of alkali-activated fly ash (class F) containing mainly Na-A-S-H gel is essentially carried out through the precipitation of alkali bicarbonate salts from the pore solution, without changing the gel structure. However, although alkali bicarbonates are soluble in water, by lowering the pH value in the first stage of carbonation, their formation partly restricts the involvement of alkali ions in alkali activation, thus reducing the efficiency of alkali activation [20]. The reaction of carbonate with calcium ion is more deleterious as it is followed by the precipitation of calcium carbonate. The pH value therefore decreases and changes the efficiency of alkali activation. Low pH retards the dissolution of reactive aluminosilicate in fly ash and mitigates the effect of polymerization on particle growth [21]. More specifically, when pH is low, the mechanism of condensation during the formation of the Gel 2 structure leads to the formation of a structure made by combining aggregation and polymerization, rather than a typical 3D structure [22]. Additionally, low pH values could also influence the increase in calcium ion concentration caused by the intensive dissolution of calcium-bearing phases in the fly ash. As a result, leached layers rich in silica are formed on the glass surface at pH values below 10–11. Dissolved calcium and magnesium later increase the pH value of the solution and further improve the alkali activation process. The dissolution process at low pH levels is characterized by incongruent dissolution because dissolved species and leached layers are formed [23]. On the contrary, dissolution at high pH levels (above 10–11) does not result in the formation of leached layers, being almost congruent to divalent metal and aluminum. Regarding the aluminum speciation, several authors [24,25] indicated a potential for aluminum species to attach to the silica surface (leached layer) when the pH of the pore solution is lower.

Besides the alkali ions present in the alkali activator, the calcium ions could also play a charge-balancing role caused by the substitution of silicate tetrahedron (SiO_4_) with (AlO_4_) in the gel structure of the alkali-activated material [26]. One potential result of concurrent alkali activation and carbonation is a change in the role of calcium ions. The increase in their concentration, caused by the acceleration of dissolution under low pH conditions [23], is expected to be followed by an increase in the pH value of the solution. This process, however, does not take place. Instead, the calcium reacts with carbonate ions to form calcium carbonate, which then precipitates and, in fact, lowers the pH of the solution. [27,28]. Therefore, the combination of consecutive processes, comprising an increased dissolution of calcium ions coupled with the formation of leached layers (incongruent reaction), as well as the expenditure of the dissolved calcium ions through the process of carbonation, could result in a reduction in pH value.

The purpose of this research is to create coarse artificial aggregates through alkali activation of Class F fly ash, which can be used in cement concrete instead of natural aggregates. In this phase of study, the influence of production methods on the characteristics of the coarse alkali-activated fly ash aggregate is examined. The mineralogical and microstructural characteristics of the samples were evaluated by means of XRD, FTIR, BSE-EDS analyses as well as through the investigation of textural properties.

## 2. Materials and Methods

### 2.1. Raw Materials

Class F fly ash (FA) from the thermal power plant “Nikola Tesla B” Obrenovac, Serbia, was used in this study as the aluminosilicate source for alkali activation. The chemical composition of the fly ash was determined by XRF (X-ray Fluorescence) analyses [29], (while reactive SiO_2_ was determined in accordance with Reference [29] and the results are presented in Table 1).

The sum of oxides (SiO_2_ + Al_2_O_3_ + Fe_2_O_3_) > 70 mass% and the loss of ignition (4.28) < mass 5% which designates used FA as class F fly ash. Using fly ash with a lower content of CaO avoids quick setting, as is the case when fly ash with a high content of calcium is used. The fineness and particle density were determined according to Reference [30]. The class of fineness was N, while particle density was 2100 kg/m^3^. Loose bulk density of FA particles was 604 kg/m^3^ [31].

### 2.2. Mix design

Fly ash was activated by an alkaline solution made of 8 M NaOH and Na-silicate with Na_2_O:SiO_2_ = 1:2 (in mass ratio) (8 M NaOH/Na-silicate = 1:2.5 mass ratio, marked as alkali activator). This mixture was prepared 24 h prior to casting/spraying. The alkaline activator/ fly ash ratio was 0.50 (in mass). The molarity of sodium hydroxide solution (8 M NaOH) was established based on literature data [32] as well as our previous work. Literature data indicates insufficient dissolution of FA for low concentrations of OH^−^ and disruption of polymerization due to high precipitation of dissolved species in premature phase when its concentration is high [33]. When 8 M NaOH solution was applied in our tests, good strength values at the age of 28 days were obtained on the samples cured in normal conditions.

### 2.3. Paste Preparation and the Creation of Aggregate by Crushing

The alkali activator was mixed with fly ash. The mixture was stirred mechanically and by hand for several minutes. It was then cast into 160 mm × 40 mm × 40 mm molds and placed on a vibrating table for 10 min to remove entrapped air. Following alkali activation under different curing conditions, the product obtained from the hardening of the alkali activated parent material was crushed.

### 2.4. Paste Preparation and the Creation of Aggregate by Pelletization

Pelletized aggregate was produced with a pelletization machine under the following conditions: The tilting angle was fixed at 39°, the mixer speed was 45 rpm, and the mixing time was up to 12 min. During pelletization, fly ash powder was poured onto the plate and continuously wetted by spraying with the alkali activator.

### 2.5. Curing Regimes and Labelling

Curing regimes were the same for both aggregates created by pelletization (VC) and aggregates created through the crushing of hardened alkali activated material (GP). A “rest period” of 24 h (the time lapse between the end of pelletization/casting and the start of curing) occurred at the beginning of all curing regimes under ambient conditions (in sealed molds for crushing and in plastic bags for pelletized aggregates, both at a temperature of 20 °C). After the rest period, samples VC 5 and GP 5 were cured under an accelerated regime (for 5 days in an oven at a temperature of 65 °C). The other samples were exposed to normal curing conditions, at a temperature of 20 °C and RH 40–60%, for 27 (VC 28, GP 28) or 119 days (VC 120, GP 120).

### 2.6. Experimental Procedures and Techniques

Phase and structural analyses were performed by X-ray diffraction and Fourier Transform Infra-Red spectroscopy methods. XRD diffractograms were recorded on a Philips PW1710 device (Amsterdam, The Netherlands) under the following experimental conditions: monochromatic Cu Kα radiation with 1.5418 Å wavelengths in the 10–55° of 2θ range, scan rate of 0.02° and 0.5 s per step, operating at 40 kV, 30 mA. Mineralogical and phase analyses were complemented by FTIR, performed on a Thermo-Nicolet Nexus 670 FTIR spectrometer (Thermo Nicolet Corporation, Madison, WI, USA; under the following experimental conditions: KBr pellet technique, spectral resolution of 4 cm^−1^, range of 1700–400 cm^−1^, 32-averaged scans per measurement) in order to obtain an integral view of the structural changes formed during the process of alkali-activated materialization. The pore size distribution (diameter range of 0.02–200 mm) and the cumulative volume of pores were determined by mercury intrusion porosimetry. Aggregate samples prepared by crushing were analyzed by an Hg Porosimeter Carlo Erba 2000 WS (Milan, Italy), while the same properties for aggregate samples obtained by pelletization were determined by a Micromeritics instrument. The results were presented as pore ratio vs. pore diameter/radius interval values. Pore ratio values were obtained by normalization of the relative cumulative volume values to the total porosity of a given sample. Pore ratio values were calculated according to Equation (1):

(1) $$Pore\;ratio=\frac{V_{p,i}}{V_{t}}\times P\left(\%\right),$$

where *V_p_*_,*i*_ is the pore volume for the specified pore radius interval, in cm^3^/g; *V_t_* is the cumulative pore volume, in cm^3^/g; and *P* is the total porosity, expressed as a percentage.

For BSE-EDS analyses (JEOL, Tokyo, Japan), the specimens were impregnated with a low viscosity epoxy resin polished with a cloth and diamond paste and then coated in gold. The EDS analyses were conducted at a magnification of 1500 and an acceleration voltage of 15 keV (software INCA 4.04, OXFORD Instruments, Abingdon, UK). Approximately 30 measurements were carried out within 3 mm of the surface. The collection time of each spectrum was 60 s. The results of EDS analyses were converted to atom proportions and then normalized to 100%.

## 3. Results and Discussion

### 3.1. Physical Properties of Aggregates

The oven-dried particle densities, as well as the apparent particle densities of aggregates and water absorption were determined on the fraction 4/8 mm in accordance with Reference [33], Table 2. Within a specified type of aggregate, the highest values for water absorption were obtained for VC 28 and GP 5 samples, whose total porosities were also the highest, Table 2.

### 3.2. Mineralogical and Structural Characterization of FA and Alkali Activated Fly Ash Aggregates

**Figure 1:**
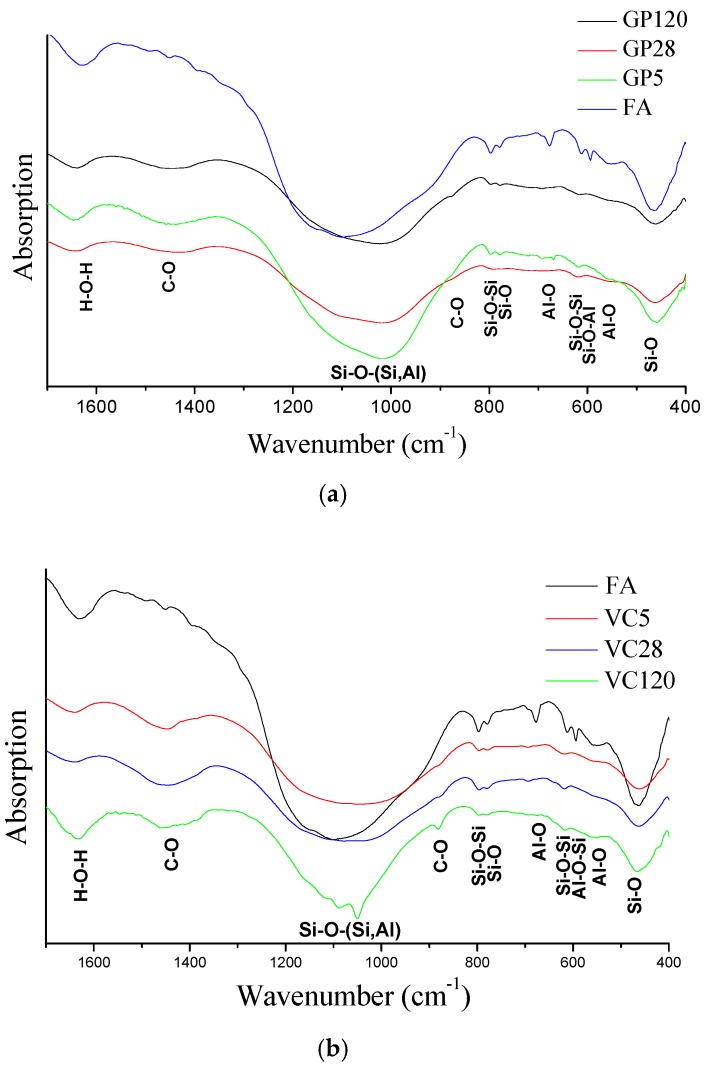
FTIR spectra of (**a**) FA and GP samples, and (**b**) FA and VC samples.

The main purpose of the FTIR technique in this paper was to study the structural evolution of amorphous alumosilicates, changes in the alkali-activated fly ash (FA), and progress in the formation of the Gel 2 structure in the different types of aggregates (GP and VC) under different curing regimes. Special importance was therefore given to the investigation of the connectivity within the Si–O–(Si, Al) frameworks in polymerized silicates and alumosilicates, i.e., the bands between 1200 and 950 cm^−1^, via shifts in specific absorption peaks corresponding to the asymmetric stretch of the given bond towards lower wavenumbers, as shown in Figure 1. The main Si–O–(Si, Al) stretching band occurred in the FA at 1090 cm^−1^. Initially, during alkali activation, this band shifted to lower wavenumbers, indicating a collapse of the Si–O network and the formation of non-bridging oxygens (depolymerization). Additionally, the substitution of a Si^4+^ for an Al^3+^ occurred, causing a reduction in the T–O–T angle (T being Al or Si) and a shift in the obtained absorption band at lower frequencies due to a smaller bonding force. Finally, sodium ions from the alkaline solution were positioned in gaps within the Gel 1 structure as charge-balancing cations. They were also present in the final structure and contributed to the overall shift of the initial absorption band. The extent of the shift mostly depends on the type of fly ash, reaction time and curing conditions, while the exact position of the bands is the result of the Al/Si ratio. The shift to lower wavenumbers was higher when there was a greater amount of tetrahedrally situated Al atoms within the newly formed structure. All these occurrences result in the formation of Gel 1 [28,29,30,31,32,33,34]. As the reaction progressed, more Si–O groups in the original fly ash were detached, increasing the concentration of Si within the pore solution. This led to the polymerization and formation of a 3D structure with a higher Si/Al ratio, i.e., Gel 2 structure. This type of structure was detected as a shift in the given band towards an absorption band at higher wavenumbers than those detected in the Gel 1 structure.

All spectra related to FA, GP and VC samples had significantly different absorption bands in the wavenumber range 1700–400 cm^−1^, as shown in Figure 1. Between 950 and 1200 cm^−1^, the absorption bands were broad, indicating a disordered silicate structure. With regard to the position of the FA absorption bands, the GP aggregates (denoted as GP 5, GP 28 and GP 120) showed a shift in peak positions towards wavenumbers lower than those registered in the VC samples (denoted as VC 5, 28 and 120). VC samples appeared to be much closer to the peak position detected in the parent fly ash structure (1090 cm^−1^). The VC 120 sample in the above-mentioned wavelength range had a sharper band with greater spectral splitting, indicating the formation of a more ordered structure.

The bands at 797 cm^−1^ and 770 cm^−1^ are respectively associated with Si–O and Si–O–Si molecular vibrations and correspond to the presence of quartz in the original fly ash. The absorption band at 550 cm^−1^ is associated with the octahedral coordinated aluminum of Al–O vibrations and indicates the presence of mullite in the FA structure. All absorption bands related to the presence of an Al-based structure (mullite) vanished or almost disappeared, suggesting the incorporation of Al ions into the described structural changes during alkali-activated materialization, as shown in Figure 1. The absorption band that appeared at 1645 cm^−1^ in all samples was attributed to the presence of H–O–H bending vibration. This band indicates that water present during alkali activation was absorbed at the surface or entrapped in the pores of the alkali-activated material products [35].

Another significant structural feature observed by FTIR analysis suggests that atmospheric carbonation was common in all specimens at any age, as seen by registered absorption bands at 1440 cm^−1^ and 873 cm^−1^, ascribed to the stretching vibration of O–C–O. These spectral features confirm that the process of carbonization undoubtedly happened during alkali activation of fly ash. The shape and intensity of the absorption band appearing at 870 cm^−1^ could indicate that carbonization was more pronounced for the VC 120 sample.

**Figure 2:**
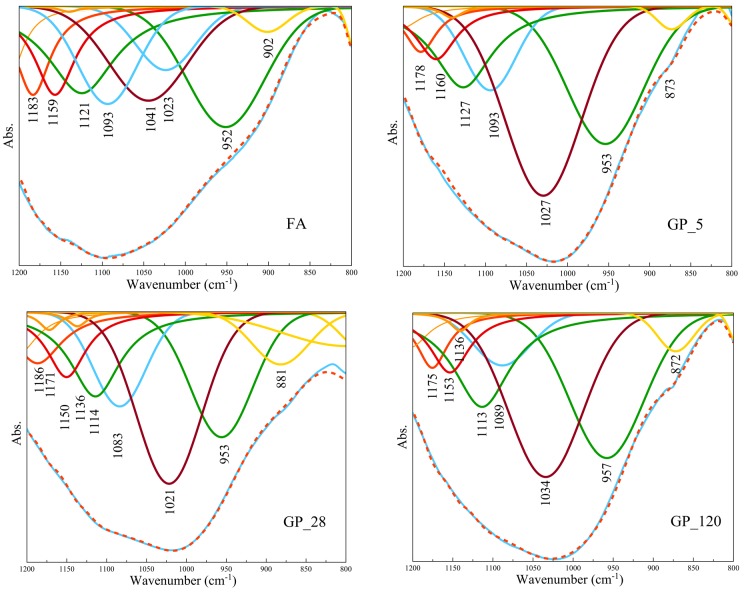

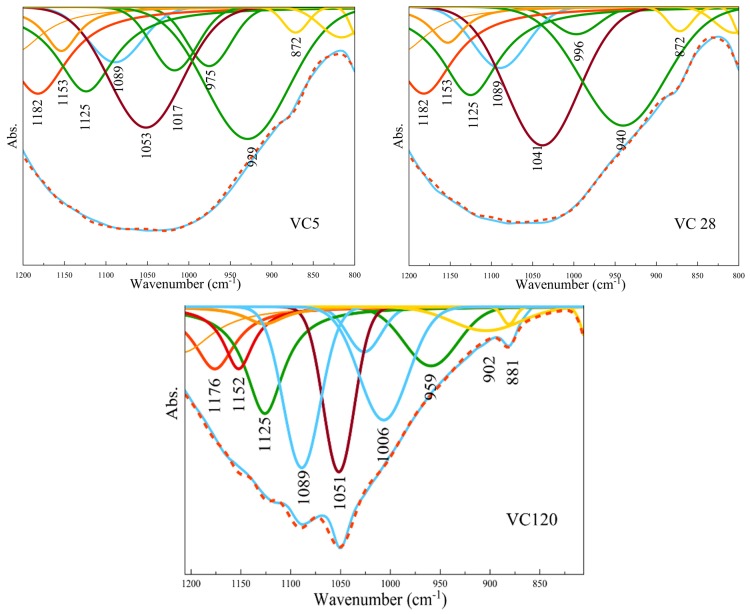
Deconvolution of Si–O asymmetric stretching band obtained by FTIR analysis. (Abs.: Absorption).

Since the spectrum bands overlap, deconvolution analyses based on Gaussian peak shape and variable peak width were applied in the wavenumber range 800–1200 cm^−1^ in order to gain a deeper insight into the number of bands not easily observable in the obtained FTIR spectra, as shown in Figure 2.

The obtained absorption bands within the given range of wavenumbers at approximately 1200, 1100, 950, 900, and 850 cm^−1^ are associated with the Si–O–Si(Al) stretching vibrations of the Q^n^ structural units where n is 4, 3, 2, 1, and 0, respectively [36,37]. Deconvolution analysis showed the prime band peaks of the parent FA at 952 cm^−1^ and 1093 cm^−1^ to be the most intensive. The position of 952 cm^−1^ peak could indicate a lower capacity for crosslinking in the amorphous phase of the parent FA, induced by increased calcium content in its structure [38]. Additionally, the peaks at wavenumbers 1041 cm^−1^ and 1093 cm^−1^ indicate the presence of a framework consisting of SiO_4_ and AlO_4_ tetrahedra in the structure of FA. The initial position of the FA peak (952 cm^−1^) remained almost unchanged during alkali activation of the fly ash in GP samples i.e., it was 953 cm^−1^ for both GP 5 and GP 28 samples and increased at 957 cm^−1^ for GP 120 sample, with a slight rise in their intensities. This rise of intensity can indicate a larger presence of the Si–O bond of non-bridged oxygens (Q^2^ structural units) [39] or could indicate the formation of C-A-S-H type gels, which implies enhanced involvement of Al^3+^ ions within the newly formed microstructures. Under different curing regimes, a number of destruction–condensation transformations took place upon activation of the Si–O–Al bonds in the FA. As a result, complex structures were formed containing mixture of Q^2^ and Q^3^ structural Si–O–Si (Al) units. Namely, the wavenumbers corresponding to the peaks in FTIR spectra changed their position from 1041 cm^−1^ in the FA to 1027 cm^−1^, 1021 cm^−1^ and 1034 cm^−1^ in the case of GP 5, GP 28 and GP 120, respectively, pointing out the occurrence of a newly formed structure upon alkali activation. The absorption wavenumbers for GP 28 and GP 120 changed their position from 1021 cm^−1^ to 1034 cm^−1^, suggesting a more progressive crosslinking and higher polymerization degree of Gel 2 type of structure over time and, consequently, an increase in Si/Al ratio. A comparison of the peak position value of thermally-treated GP 5 samples (1027 cm^−1^) with that of non-thermally treated GP 28 samples (1021 cm^−1^) revealed a shift towards lower wavenumbers, suggesting that an elevated curing temperature had a pronounced influence on the rise in polymerization level. An increase in the intensity of the deconvoluted peaks within the corresponding wavenumber range (1020–1040 cm^−1^) for GP samples presents a clear indication of a larger amount of the newly formed amorphous structure over the period of time. The main parent FA absorption peak, other than the one observed at ~950 cm^−1^, was also detected in GP samples indicating the presence of unreacted fly ash particles within the structure, as presented in Table 3.

Deconvolution analyses of VC sample spectra showed different spectral features and peaks at different positions in comparison to those for GP samples. Namely, the peak in FA at 952 cm^−1^ shifted to lower wavenumbers in VC 5 (940 cm^−1^) and VC 28 (929 cm^−1^), while in the case of VC 120, it shifted toward higher wavenumbers (959 cm^−1^). It indicates significant changes of Si–O–Si structural units (Q^2^) as opposed to GP samples, especially emphasizing the stronger influence of alkali cations within the newly developed structure upon alkali activation performed by the pelletization method of preparation. This is highlighted in the case of VC 28 samples, which retain the highest deflection of absorption peak position. It designates a noticeable influence of curing temperature on structural changes. Namely, it appears that due to a higher temperature, a pronounced dissolution at an early age took place enabling the increase of the availability of hydrolyzed aluminoslicates and silicate species. This asymmetric stretching vibration of Si–O could indicate the formation of C-A-S-H type gels which designate enhanced involvement of Al^3+^ ions unlike that in the case of GP samples. This was distinguished on deconvoluted FTIR spectra as a shift of the given peak towards a lower wavenumber region. Gel 1 structure probably remained due to the lack of conditions for transformation into a Gel 2 structure [40]. Additionally, the absorption peaks at 1017 cm^−1^, 996 cm^−1^ and 1006 cm^−1^ in deconvoluted FTIR spectra of VC 5, VC 28 and VC 120, respectively, suggest both a higher involvement of alkali ions upon alkali activation than in GP samples and the formation of a mixture of Gel 1 and Gel 2 structures which was followed by a lower polymerization degree with lower Si/Al ratio in comparison to GP samples. The absorption peak of FA at 1041 cm^−1^ shifted to higher wavenumbers, i.e., 1053 cm^−1^ in the case of VC 28 and 1051 cm^−1^ in the case of VC 120, while in the case of VC 5 it remained the same as FA, as shown in Figure 2. There are several possible explanations for the fact that the absorption peaks corresponding to the molecular vibration of the Si–O–Si (Al) structure had higher wavenumber values, closer to the peak positions found in the case of the parent FA structure. On one hand, this could be interpreted as the presence of a higher amount of the polymerized amorphous structure (samples rich in Si compound exhibit a higher Si/Al ratio); on the other hand, these bands could be attributed to the stretching of Si–O–Si bonds which are present as unreacted silica particles, originating either from unreacted FA or from the alkaline activator. This observation should be further checked through SEM-EDS analyses [41].

Additionally, absorption peaks at around 880 cm^−1^ and 1180 cm^−1^ are characteristic of both types of aggregate and may correspond to the role of Mg which is incorporated into the Si–O bond [42] and bridges Si–O bonds in the crystalline phases [43]. Moreover, the main absorption band for the reaction products is placed at around 940–960 cm^−1^ and is attributed to the asymmetric stretching vibration of Si–O terminal bonds [43,44]. This is a typical band that indicates the formation of C-A-S-H type gels with chain assemblies within the newly formed microstructures. Furthermore, the absorption bands observed in both types of aggregate (GP and VC) at ~870 cm^−1^, 1020–1035 cm^−1^ and ~1090 cm^−1^, respectively correspond to the stretching vibration of Si–O–Al, the Si–O–Si (Al) tetrahedra and the remains of the original fly ash.

**Figure 3:**
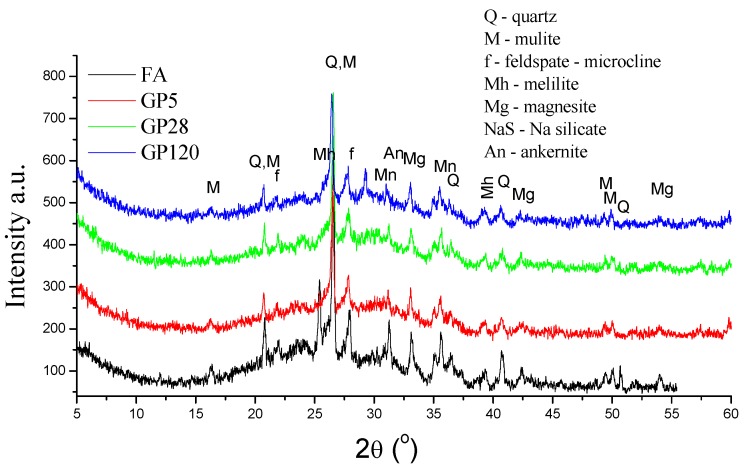
XRD diffractograms of FA and GPs.

**Figure 4:**
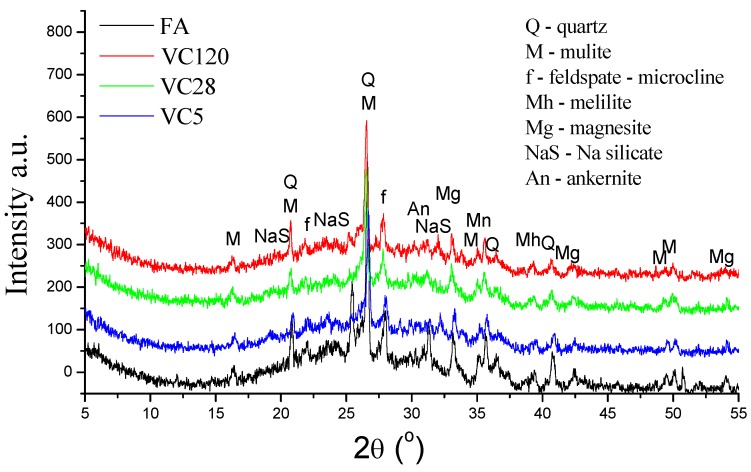
XRD diffractograms of FA and VCRs.

A comparison of the mineralogical composition of the original as-received FA and the two types of aggregate prepared by different granulation methods is shown in Figure 3 and Figure 4. The XRD analyses showed that, regardless of the curing method, alkali-activated fly ash has an amorphous to semi-crystalline structure and contains crystalline phases. The latter includes quartz, mullite, feldspar-like minerals, maghemite, magnetite, and magnesite. The broad hump located between 17° and 35° of 2θ values is due to the presence of the amorphous/glassy phases in the FA. On the other hand, a similar hump in the alkali-activated fly ash aggregate represents the remaining unreacted particles of the amorphous phase that partially overlapped with sodium aluminosilicate products, i.e., feldspar-like mineral phase. Following alkali activation of the fly ash, the original broad peak of the FA shifted to slightly greater values of 2θ angles, indicating dissolution of the fly ash amorphous phase and the formation of a new amorphous phase in the alkali-activated fly ash aggregates. Besides the change in the broad hump, a decrease in the intensity of mullite peaks suggests its involvement in the alkali activation process, which was confirmed by FTIR analysis. Additionally, these results indicate a slight decrease in the peak intensities related to melilite, magnesite and feldspar-like minerals, suggesting they are also involved in the fly ash alkali activation process. Furthermore, FTIR analysis indicated that the main structural changes occurred in the amorphous phase.

### 3.3. BSE-EDS Analysis

**Figure 5:**
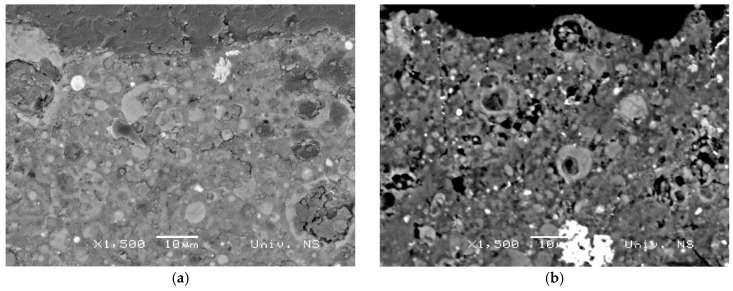
BSE micrograph of samples (**a**) GP 28 and (**b**) VC 28.

**Figure 6:**
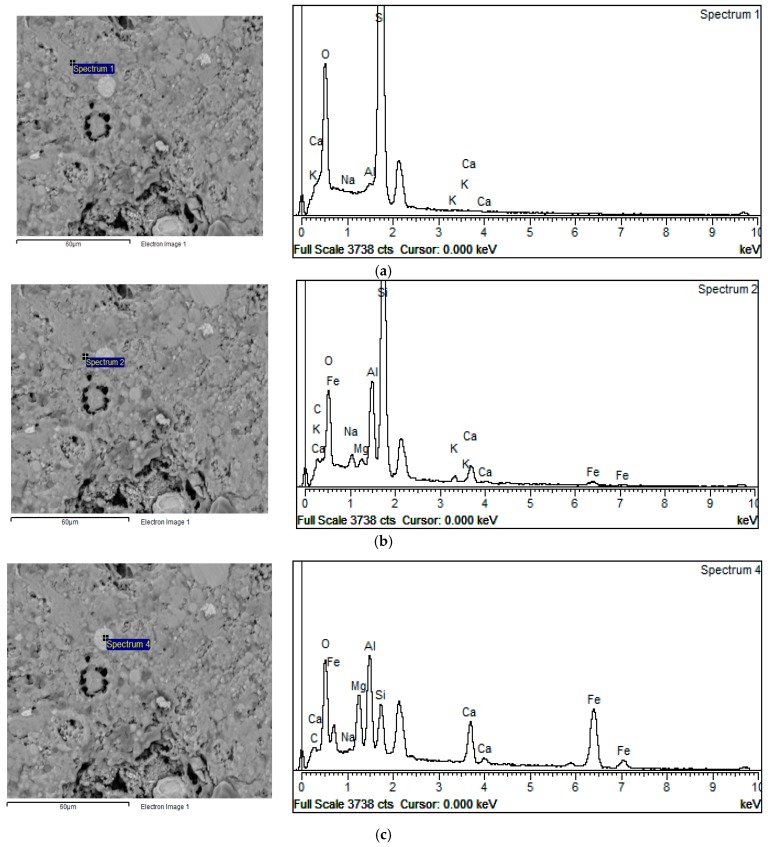
BSE micrograph of sample VC 120, (**a**) silica phase, (**b**) alumino-silicate phase, (**c**) predominantly aluminum phase.

Information obtained from sample surface analysis using the BSE-EDS characterization technique can reveal important compositional changes induced by differences in the initial settings of alkali activation of fly ash chemistry and, therefore, provide valuable information about the com-position of Gel 2 [12,26,45,46]. According to the results presented in Figure 5, an inhomogeneous alkali-activated fly ash structure was obtained for both types of sample (VC 28 and GP 28). Similar results were obtained for all other samples. In general, incompletely dissolved grains in the alkali-activated fly ash matrix and the phases developed during the incongruent dissolution of parent material are visible. These results are in accordance with FTIR results related to the registered absorption bands of unreacted grains of fly ash in the alkali-activated fly ash samples. Figure 6 shows VC 120, which is representative of incongruent dissolution. This was observed through the formation of phases characterized by differences in composition. Spectrum 1, for instance, mainly indicates the presence of a silica phase, whereas spectrum 2 shows the presence of an alumino–silicate phase and spectrum 4 presents a clearly separated area containing predominantly aluminum (Figure 6). The presence of separated, predominantly silica and alumina phases (spectra 1 and 4) indicates that polymerization was not complete, i.e., aluminum and silicon species released during dissolution were not incorporated into the polymer structure. These results are in consent with the results obtained by FTIR analysis. It can therefore be concluded that the Gel 2 structure was probably formed at a low pH [21].

**Figure 7:**
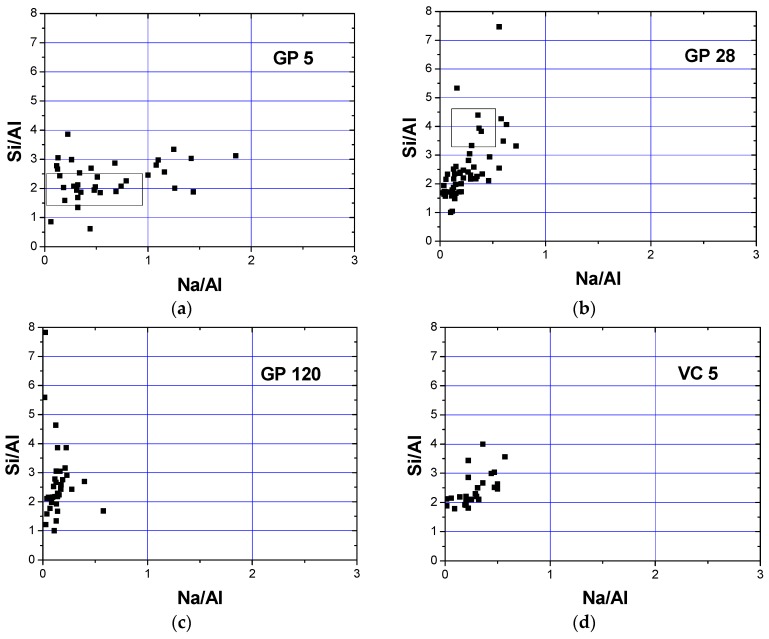

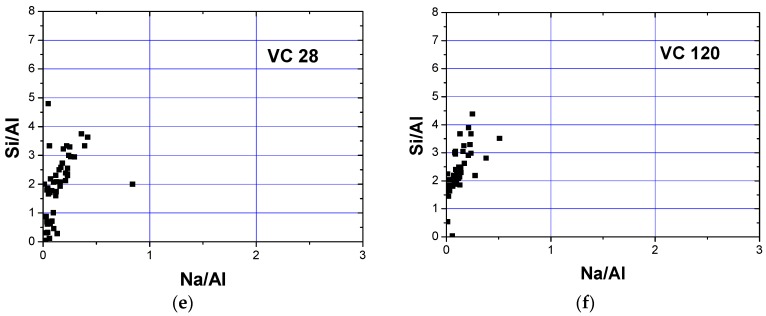
Atomic ratios Si/Al vs. Na/Al for (**a**–**c**) GP and (**d**–**f**) VC aggregates.

FTIR analysis registered carbonation for all samples while calcium was recorded by EDS analyses (Figure 6). This calcium might originate from calcium carbonate or from portlandite. Conversely, XRD analyses did not confirm the presence of portlandite or calcium carbonate in crystalline form. The formation of the Gel 2 structure is characterized by the condensation of chemical species, leading to the formation of a colloid with a 3-D structure [47]. Na^+^ plays an important role in this structure since it enables a balance in electrical charge when Al^3+^ ions replace Si^4+^ ions. If aluminum ions are included in the 3-D structure, an equivalent number of Na^+^ ions are also included. To assess this process, it is useful to follow the changes in the Na/Al ratio. The stoichiometric value of this ratio is 1 [21]. The atomic ratios of Si/Al vs. Na/Al are illustrated in Figure 7, with a range of values clustered around one or two distinct regions being attributed to the formation of different phases during alkali activation. Figure 7 shows the influence of curing conditions on GP (a–c) and VC (d–f) aggregates. In the case of both types of aggregate, the atomic ratios of Si/Al were distributed in a wider cluster when the samples were treated at an elevated temperature, denoting that phases with a greatly varied Si/Al ratio (1.5 ≤ Si/Al ≤ 3) were formed after alkali activation. Moreover, the Na/Al ratio was in the range 0 ≤ Na/Al ≤ 0.75 in VC 5 samples and 0.2 ≤ Na/Al ≤ 1.5 in GP 5 samples (Figure 7a,d). In the case of GP 5, a large number of cluster points have a Na/Al ratio close to 1, indicating a stoichiometric reaction occurred during alkali activation. Some values of the Na/Al ratio were higher than 1, which could point to some phases being rich in sodium, most likely due to the presence of an unreacted alkali activator [15]. On the other hand, in VC 5 samples the Na/Al atomic ratio was less than 1 (Na/Al ≈ 0.25), implying that the amount of sodium given as a charge balancing cation was insufficient during the formation of the gel structure. A low concentration of Na^+^ ion has also been documented in other research [48]. Shi reported that at a low pH, an ion-exchanging process took place in N-A-S-H gel in which natrium was replaced by calcium [26]. The lower Na/Al ratio in VC 5 samples implies that a partial integration of calcium into the gel structure was necessary in order to achieve charge balance. Calcium would certainly have been available had a lower pH value been obtained during pelletization due to carbonation. Additionally, an increased alkali activator/ fly ash ratio occurred on and near the outer surface (compared to the bulk of the sample), which could have affected the degree of fly ash activation and dissolution. These facts imply that the content of dissolved Ca^2+^ ions could be considerably higher in VC 5 samples than that in GP 5, although the quantity of Ca^2+^ was not significant in the original fly ash (below 7 mass%, Table 1). Both samples (GP 5 and VC 5) showed two distinct clusters in the Si/Al ratio: the first cluster grouped around an atomic ratio of Si/Al ≈ 2 and the other with a considerably higher silicium content which appears to be far closer in composition to the atomic ratio of the precursor (Si/Al = 3.0).

The atomic composition, with reference to the atomic ratios of Si/A1 vs. Na/A1, was within a narrower range in samples exposed to normal curing conditions for 28 and 120 days (VC 28, VC 120 and GP 28, GP 120) than it was for the samples exposed to 65 °C. According to Xie [14], one of the beneficial roles of heating could be seen through the acceleration of water evaporation during the polymerization stage i.e., during the formation of the Gel 2 structure. Thus, abundant water is favorable for the stages of dissolution and hydrolysis but detrimental for polymerization and formation of the Gel 2 structure. The atomic ratios of Si/Al vs. Na/Al for samples VC 28, VC 120 and GP 28 are mostly grouped in two clusters (Figure 7). The first cluster is in the range 1.5 < Si/Al < 2.5, while the second equals Si/Al = 3. In these cases, the value of the atomic ratio Na/Al falls below 0.25. Additionally, the atomic ratio values for VC 28 were in the ranges of a high content of aluminum (0 < Si/Al < 1, 0 < Na/Al < 0.25), indicating that Al-rich secondary phases were formed. The cluster regions after 28 and 120 days of curing are attributed to a mixture of dealuminated residual fly ash particles and an N-A-S-H gel structure of variable Si/Al ratio. The regions rich in Al are likely to represent an intermixture of the residual mullite-like type of glassy phase (confirmed by XRD analysis) and a certain amount of N-A-S-H gel structure, especially in the case of the GP aggregate samples. N-A-S-H gels formed in GP samples are characterized by a higher Si content than in VC samples, resulting in a less pronounced need for Na^+^ as a charge balancing element in the alkali-activated fly ash network.

**Figure 8:**
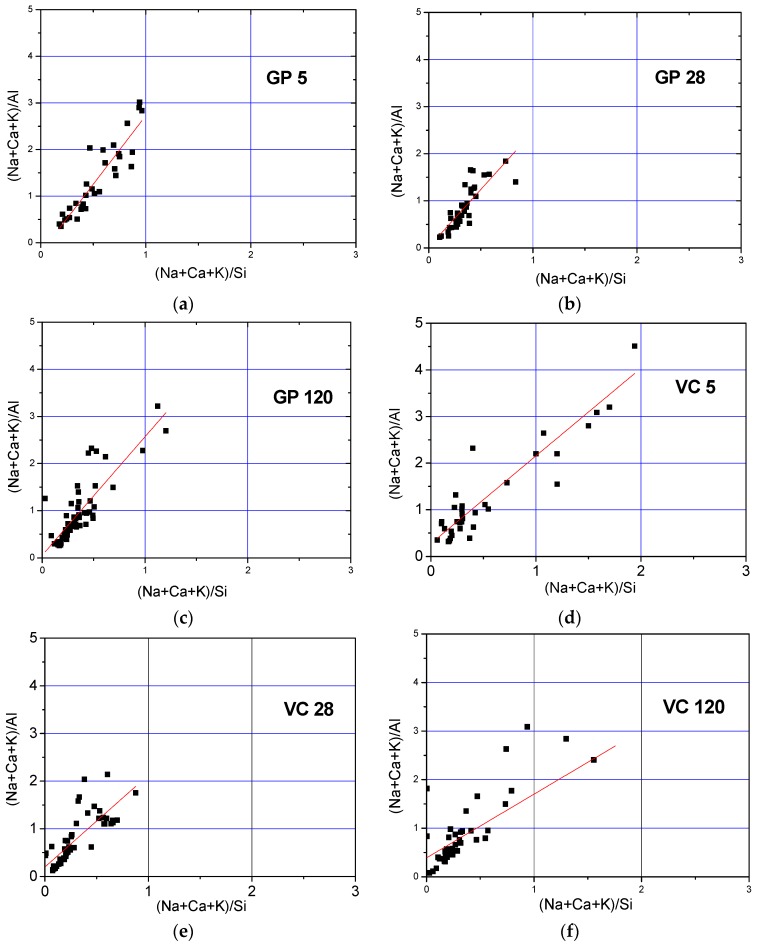
Atomic ratios (Na + Ca + K)/Al vs. (Na + Ca + K)/Si for (**a**–**c**) GP and (**d**–**f**) VC aggregates.

In order to investigate the correlation between the Si/Al ratio and the involvement of Ca, K, and Na alkalis in the structure of the developed alkali-activated material, the atomic ratios (Na + Ca + K)/Al vs. (Na + Ca + K)/Si are presented in Figure 8. In the case of VC 5 and GP 5, the atomic ratio of (Na + Ca + K)/Al appears to be relatively higher than 1, while a much tighter cluster distribution is observed following an increased curing time, roughly within stoichiometric boundaries. The slope of the least squares fitted trend lines gives an indication of the Si/Al atomic ratio values, i.e., the development of the Gel 2 structure. Table 4 outlines the slope values, which are higher for GP samples (obtained by the crushing method) than for VC samples (obtained by the pelletization method). It is therefore indicated that the method of aggregate production influenced the formation of different paths of fly ash alkali activation and, subsequently, the alkali-activated fly ash structure.

The lower atomic ratio of Si/Al observed in the VC samples, as shown in Table 4, could either be the result of a higher activator/fly ash ratio on the granule surface, indicating an increase in silicon concentration, or a decrease in pH value due to the process of carbonation, and the consequent increase in dissolution of calcium from calcium-bearing phases in fly ash. The increased dissolution of calcium would affect the carbonation process and pronounce the negative effects even further. According to Duxson et al. [26], the increase in silicon concentration obtained in the case of a higher activator/fly ash ratio value at the pellet surface retards the dissolution rates of alumino-silicate minerals in the parent fly ash. Later on, values of Si/Al ratio higher than 1 will allow the integration of the majority of aluminum into the alumino–silicate oligomers that could be used for polymerization. In other words, the final value of the Si/Al ratio will be influenced by the amount of dissolved aluminum and the concentration of silicon in the initial solution [49].

As reported by P. Duxson at al [26], the mechanism of aluminum incorporation into the structure of the alkali-activated material i.e., formation of the Gel 2 structure, is highly dependent on the composition of the silicate activator. This study suggests that for low ratios of Si/Al (Si/Al < 1.4), the aluminum released during dissolution was partly incorporated into the alkali-activated fly ash matrix. On the other hand, alkali-activated fly ash synthesized by an alkali activator comprising a higher content of silicon, and consequently a higher Si/Al ratio (Si/Al > 1.6), will enable the incorporation of a substantial amount of dissolved aluminum into the aluminosilicate oligomers which are consumed upon alkali activation and the formation of Gel 2. The observed results in the given references could offer an explanation for the results obtained in our study i.e., a low Si/Al ratio for the aggregate obtained by pelletization (VC samples). Specifically, the initial concentration of silicon close to the surface was higher in the aggregate obtained by pelletization than that produced via the crushing method, due to the method of application of the alkali activator. The increased ratio of the alkali activator/fly ash on the surface could be of importance to the incorporation of dissolved aluminum into the Gel 2 structure. On the other hand, the presence of separated, predominantly silica and alumina phases (spectra 1 and 4), as shown in Figure 6, indicates that the abovementioned speculations are not the prevailing cause of the low Si/Al atomic ratio in VC samples.

**Figure 9:**
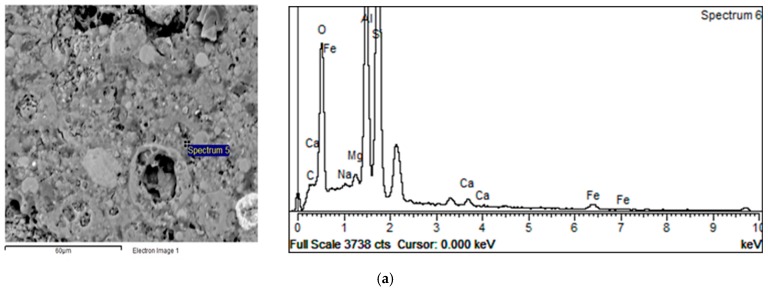

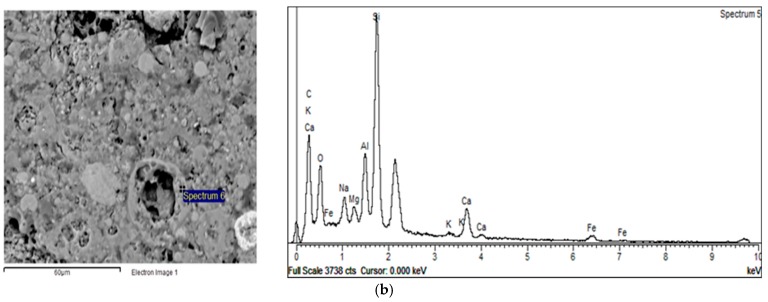
BSE-EDS analyses of the VC 120 sample; (**a**) dissolved part of a parent fly ash particle and (**b**) particle shell richer in Al.

Furthermore, in the case of aggregate production through pelletization, other phenomena such as carbonation and the more intensive evaporation of water [16] could have also influenced the formation of the Gel 2 structure. Specifically, these two phenomena influence the pH of an alkaline solution. Carbonation decreases the pH value, while the evaporation of water increases the concentration of all species in the solution, thus also changing the pH value. A detailed evaluation of the influence of these phenomena was not possible in this study. Figure 9 presents the differences in atomic composition between the dissolved part of a parent fly ash particle, Spectrum 5, and a particle shell which is richer in Al (Figure 9, spectrum 6). These results suggest that alumina was attached to the silicium-rich leached layer which remained following the fly ash dissolution.

### 3.4. Textural Properties

The investigation of textural properties showed obvious differences between the two types of aggregates. Table 5 shows that a higher total porosity was observed in the samples produced by pelletization (VC 28 and VC 120) as compared to the samples obtained by the crushing method (GP 28 and GP 120). The exception was VC 5, the samples treated at an elevated temperature. Overall, in both types of aggregate, the total porosity values (Table 5) were lower in the samples cured at room temperature for 28 and 120 days in comparison to those cured at 65 °C. In the case of samples created via the crushing method, porosity decreased from 36.1%, through 17.4% to 12.93% (GP 5, GP 28 and GP 120, respectively), while in pelletized samples it declined from 34.61% to 29.15% from VC 5 to VC 120. The value for VC 28 was 40.22%. The higher total porosity of samples exposed to an elevated temperature might be caused by the development of water vapor pressure during the curing process. On the other hand, the reduction in total porosity in both aggregate types between 28 and 120 days of curing (Table 5) could be the consequence of polycondensation, followed by the upraise and exit of water accompanied by a decrease in capillary porosity [18]. The results obtained by Bernal et al. [50] suggest that the carbonation of alkali-activated materials increases both total porosity and the volume of permeable pores, which is not typical of porosity results obtained when cement is used as a binder. The increased carbonation of VC aggregates might also therefore trigger the increase in porosity.

**Figure 10:**
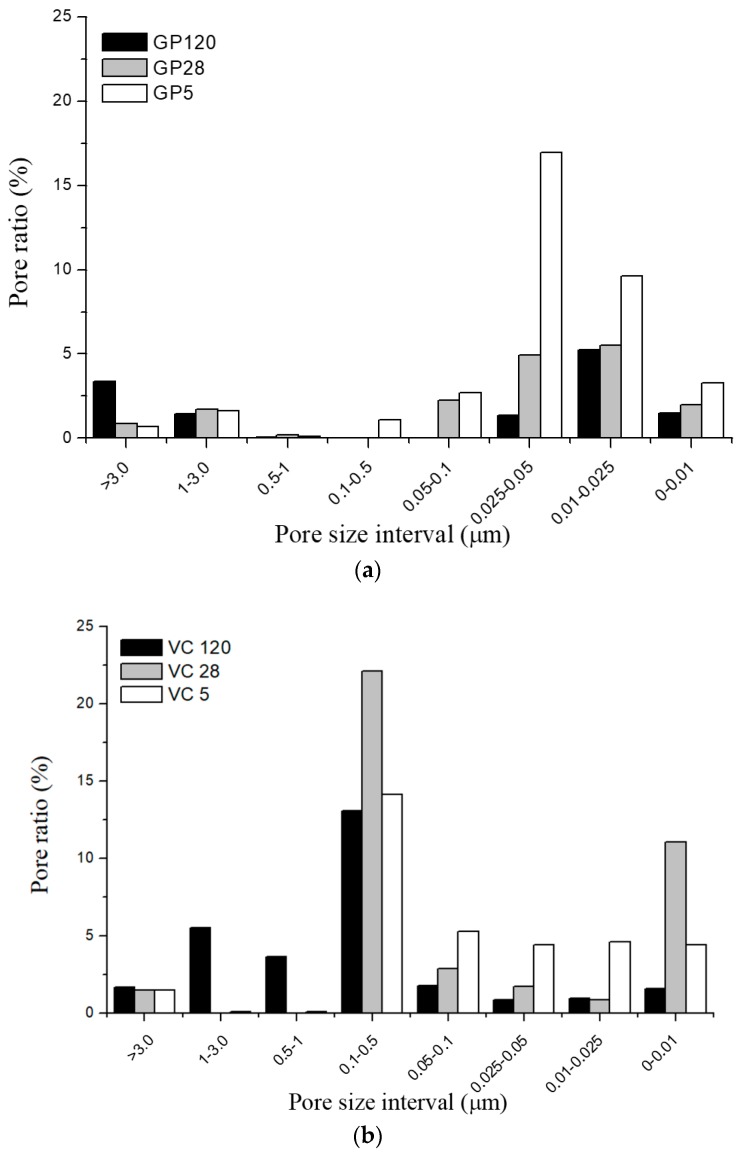
Pore size distribution of (**a**) GP and (**b**) VC aggregates.

Although the differences in total porosity between the samples cured at 65 °C for 5 days and the samples cured at room temperature for 28 and 120 days showed a similar course for both types of aggregate, the pattern for pore size distribution (PSD) was quite different, as shown in Figure 10. The PSD observed in GP samples was approximately 0.01–0.05 µm, while in VC samples, PSD was predominantly in the range of 0.1–0.5 µm. The highest ratio of capillary pores with a radius below 0.1 µm was found in VC 5 and GP 5, while the highest number of large pores (with a radius over 1 µm) occurred in GP 120 and VC 120. The differences observed in PSD were the result of textural rearrangements developed during alkali activation of the fly ash, caused by differences in the mechanisms of alkali activation. Basically, the porosity developed during this process is presumed to be a result of the polymerization process which is connected to the formation of the Gel 2 structure.

The polymerization of aluminosilicate oligomers and formation of the Gel 2 structure is represented by the continued formation of links between oligomers, followed by the expulsion of water into larger pores [49]. The higher the value of the Si/Al ratio, the greater the extent of polymerization i.e., the structure contains a greater amount of gel. In this structure, the amount of expelled water is higher. This water will settle into the pores slightly larger in diameter than those in Gel 2 if the process of its ejection into larger pores is hindered or too slow. P. Duxson et al. [26,49] reported that an increase in the Si/Al ratio, i.e., silica content, leads to the formation of smaller pores containing water and excess hydroxide. These small pores are formed within densely packed globular polymeric entities [49], while some of the water is transported into larger pores.

BSE-EDS analyses of GP samples revealed a higher Si/Al ratio, i.e., a greater volume of gel, in comparison to VC samples. The PSD was therefore shifted to lower pore diameters (less than 0.05 μm) for GP samples, whereas VC samples had dominantly larger pores, in the range of 0.1–0.5 μm. Examining the influence of curing conditions on PSD revealed that exposure to an elevated temperature leads to a higher ratio of pores smaller than 0.5 μm in both types of aggregate. On the other hand, curing in normal conditions for 120 days leads to the creation of larger pores. These results demonstrate that the processes of structural rearrangement are dependent on the curing regime and are not finished even when the sample is hardened.

## 4. Conclusions

The aggregates prepared by the alkali activation of Class F fly ash, using two different granulation processes (crushing and pelletization) and cured under different regimes (at a temperature of 65 °C for 5 days and under normal conditions at a temperature of 20 °C, and RH 40–60% for 28 and 120 days), exhibited various mineralogical, structural and textural properties. The different granulation methods caused different mechanisms which resulted in diverse structural features:

- FTIR analyses showed that the greatest changes occurred in the amorphous phase in both types of aggregate. FTIR analyses indicated a better dilution of fly ash in the aggregate obtained by crushing compared to the aggregate obtained by pelletization of what was confirmed by the changes in FTIR peak positions. Polymerization was most pronounced for the aggregates exposed to 65 °C for 5 days.
- FTIR analyses also showed that the process of carbonation occurred upon alkali activation across all investigated aggregate samples, this being more pronounced in the case of the aggregate obtained by pelletization in comparison to the aggregate obtained by the crushing method. Since the process of carbonation decreases pH value, it is presumed that the N-A-S-H structure of Gel 2 was probably formed at a low pH.
- The XRD analyses showed that neither the granulation method nor the curing conditions had a great influence on crystal phase composition. BSE-EDS analyses indicated that the structures obtained by pelletization contained phases developed during the incongruent dissolution of the parent material (silica and alumina phases), in addition to incompletely dissolved grains from the alkali-activated fly ash matrix.
- The Si/Al atomic ratio was greater in the aggregate obtained by crushing than in that formed by pelletization. The lower Si/Al atomic ratio observed in the VC samples could be the result of two things: a higher activator/fly ash ratio value on the granule surface, indicating an increase in silicon concentration, and the decrease in pH value due to carbonation.
- The total porosity values were lower for the samples cured in normal conditions for 28 and 120 days in comparison to those cured at 65 °C. Pore size distribution was correlated to the amount of gel and the curing regime; exposure to an elevated temperature led to a higher ratio of pores smaller than 0.5 μm in both types of aggregates. Conversely, curing under normal conditions for 120 days led to the creation of larger pores.

## Figures and Tables

**Table 1:** Chemical composition of FA (mass%)**.**

SiO_2_	Al_2_O_3_	Fe_2_O_3_	SO_3_	CaO	MgO	Na_2_O	K_2_O	Cl^−^	SiO_2_r ^1^	LOI
56.1	19.7	5.36	3.1	6.95	1.83	0.60	2.05	0.007	37.34	4.28

^1^ SiO_2_r—reactive silica.

**Table 2:** Oven dried particle density, apparent particle density and water absorption of crushed and pelletized aggregates.

Aggregate	Oven Dried Particle Density, kg/m^3^	Apparent Particle Density, kg/m^3^	Water Absorption after 24 h, %
GP 5	1495	2585	28.2
GP 28	1490	2526	27.5
GP 120	1492	2530	26.0
VC 5	1470	2400	26.4
VC 28	1348	2570	35.3
VC 120	1450	2350	26.5

**Table 3:** Absorption peak values of the deconvoluted FTIR spectra.

FA	GP 5	GP 28	GP 120	VC 5	VC 28	VC 120
Absorption peak position (cm^−1^)						
	873	881	872	872	872	881
902						902
952	953	953	957	929	940	959
				975		
1023	1027	1021	1034	1017	996	1006
1041				1053	1041	1051
1093	1093	1083	1089	1089	1089	1089
1121	1127	1114	1113	1125	1125	1125
		1136	1136			
1159	1160	1150	1153	1153	1153	1152
		1171				
1183	1178	1186	1175	1182	1182	1176

**Table 4:** Slope values of the linear curve presented in Figure 8.

Sample	Slope of the Linear Curve Si/Al
GP 5	2.94
GP 28	2.41
GP 120	2.51
VC 5	1.88
VC 28	1.93
VC 120	1.95

**Table 5:** Total porosity of GP and VC aggregates.

Sample	Total Porosity (%)
GP 5	36.1
GP 28	17.4
GP 120	12.9
VC 5	34.6
VC 28	40.2
VC 120	29.2
